# (*E*)-2-(2-Fluoro­benzyl­idene)butanoic acid

**DOI:** 10.1107/S1600536808007149

**Published:** 2008-03-20

**Authors:** Muhammad Niaz, M. Nawaz Tahir, Zia-ur-Rehman, Saqib Ali, Islam Ullah Khan

**Affiliations:** a Department of Chemistry, Quaid-i-Azam University, Islamabad 45320, Pakistan; b Department of Physics, University of Sargodha, Sagrodha, Pakistan; c Department of Chemistry, Government College University, Lahore, Pakistan

## Abstract

In the crystal structure of the title compound, C_11_H_11_FO_2_, the methine CH forms an intra­molecular hydrogen bond with the carboxyl­ O atom. The mol­ecules form dimers through hydrogen bonding between carboxyl­ groups. These dimers are linked to each other by C—H⋯O contacts between the benzene and carbonyl groups of adjoining mol­ecules. In addition, there are weak inter­molecular C—H⋯F contacts.

## Related literature

For related literature, see: Burns & Hagaman (1993 ▶); Burt (2004 ▶); Forgó *et al.* (2005 ▶); Hertog *et al.* (1995 ▶); Muhammad *et al.* (2007 ▶). For details of the Cambridge Structural Database, see: Allen (2002 ▶).
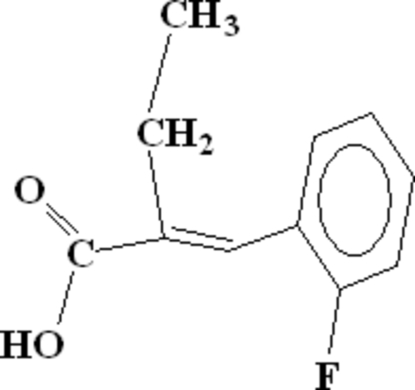


## Experimental

### 

#### Crystal data

C_11_H_11_FO_2_
*M* *_r_* = 194.20
Monoclinic, 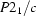

*a* = 4.1895 (4) Å
*b* = 17.4362 (19) Å
*c* = 13.8134 (15) Å
β = 96.719 (3)°
*V* = 1002.12 (18) Å^3^
*Z* = 4
Mo *K*α radiation radiation
μ = 0.10 mm^−1^
*T* = 296 (2) K
0.25 × 0.18 × 0.12 mm

#### Data collection

Bruker Kappa APEXII CCD diffractometer
Absorption correction: multi-scan (*SADABS*; Bruker, 2005 ▶) *T*_min_ = 0.935, *T*_max_ = 0.958
8632 measured reflections
2981 independent reflections
1704 reflections with *I* > 2σ(*I*)
*R*_int_ = 0.026

#### Refinement

*R*[*F* ^2^ > 2σ(*F* ^2^)] = 0.050
*wR*(*F* ^2^) = 0.164
*S* = 1.04
2981 reflections
131 parameters
H atoms treated by a mixture of independent and constrained refinement
Δρ_max_ = 0.41 e Å^−3^
Δρ_min_ = −0.21 e Å^−3^

### 

Data collection: *APEX2* (Bruker, 2007 ▶); cell refinement: *APEX2*; data reduction: *SAINT* (Bruker, 2007 ▶); program(s) used to solve structure: *SHELXS97* (Sheldrick, 2008 ▶); program(s) used to refine structure: *SHELXL97* (Sheldrick, 2008 ▶); molecular graphics: *ORTEP-3 for Windows* (Farrugia, 1997 ▶) and *PLATON* (Spek, 2003 ▶); software used to prepare material for publication: *WinGX* (Farrugia, 1999 ▶) and *PLATON*.

## Supplementary Material

Crystal structure: contains datablocks global, I. DOI: 10.1107/S1600536808007149/bv2091sup1.cif

Structure factors: contains datablocks I. DOI: 10.1107/S1600536808007149/bv2091Isup2.hkl

Additional supplementary materials:  crystallographic information; 3D view; checkCIF report

## Figures and Tables

**Table 1:** Hydrogen-bond geometry (Å, °)

*D*—H⋯*A*	*D*—H	H⋯*A*	*D*⋯*A*	*D*—H⋯*A*
O1—H1⋯O2^i^	0.97 (2)	1.66 (2)	2.6325 (18)	177.7 (12)
C3—H3⋯O1	0.93	2.32	2.713 (2)	105
C6—H6⋯O2^ii^	0.93	2.53	3.421 (2)	160
C8—H8⋯F1^iii^	0.93	2.55	3.266 (2)	134 (1)

Symmetry codes: (i) 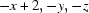; (ii) 
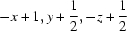
; (iii) 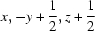.

**Fig. 1.**
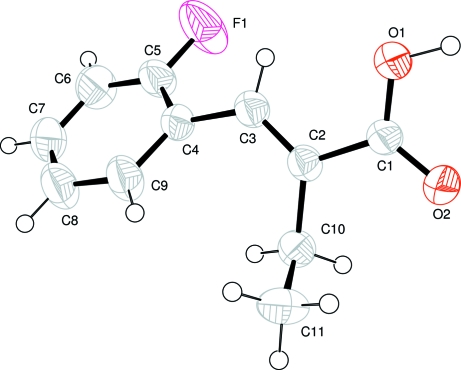
ORTEP drawing of the title compound, C11H11F1O2 with the atom numbering scheme. The thermal ellipsoids are drawn at the 50% probability level. H-atoms are shown by small circles of arbitrary radii.

**Fig. 2.**
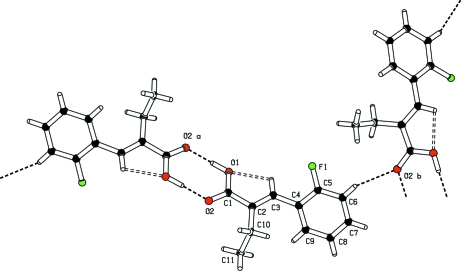
The packing figure (PLATON: Spek, 2003) which shows the dimeric nature of the compound owing to inter molecular hydrogen bonding and also showing a link between dimers.

## supplementary crystallographic information

### Comment

Cinnamic acid derivatives are well known for their antibacterial, antifungal antiparasitic (Burt, 2004), and antitumor activity (Hertog *et al.*, 1995). They are also used in the shikmic acid metabolic pathways of higher plants (Forgó *et al.*, 2005).

In the structure of the title compound (I), there are two C-atoms between the carboxylate and 2-fluorophenyl C-atoms. A search of CCDC (Allen, 2002) shows that a structure of 2-amino-2-(2-fluorophenyl)acetic acid (Burns *et al.*, 1993) has been published in which there is only a single C-atom between carboxylate and phenyl ring. Moreover, there is no structure of this kind with a different position for the F-atom.

The C1=O2 bond distance [1.2301 (18) Å], is significantly shorter than the C1—O1 distance [1.3006 (18) Å]. The C1—O1 bond lengthened due to the formation of intramolecular and intermolecular H-bonds. The value of C2=C3 is 1.334 (2) Å. The phenyl ring bond distances are in the normal range but the C4—C5—C6 bond angle is 124.27 (18)°, due to the influence of the F substituent attached to C5. The dihedral angle between the planes formed by (O1, C1, and O2) and (C2, C10, and C11) is 80.97 (18)°, and the dihedral angles between these planes and the phenyl ring are 52.88 (10)° and 67.17 (15)° respectively. The molecules are stabilized by intramolecular and intermolecular H-bonds. The title compound forms dimers through H-bonding, O1—H1···O2^i^ [symmetry code i = -*x* + 2, -*y*, -*z*] as shown in Fig 2. These dimers are linked to each other through a C6—H6···O2^ii^ interaction [symmetry code ii = -*x* + 1, *y* + 1/2, -*z* + 1/2]. Details of the H-bonding are given in Table 1. In addition there is a weak C8—H8···F1^iii^ intermolecular interaction [symmetry code iii = *x*, 1/2 - *y*, 1/2 + *z*] with a distance 3.2658 (25) Å between C8 and F1^iii^.

### Experimental

Compound (I) was synthesized as reported earlier (Niaz, *et al.*, 2007). A mixture of 2-fluorobenzaldehyde (1.05 ml, 10 mmol), ethylmalonic acid (2.64 g, 20 mmol) and piperidine (1.98 ml, 20 mmol) in a pyridine (12.5 ml) solution was heated on a steam-bath for 24 h. The reaction mixture was cooled and added to a mixture of 25 ml of concentrated HCl and 50 g of ice. The precipitate formed in the acidified mixture was filtered off and washed with ice-cold water. The product was recrystallized from ethanol. The yield was 65%, m.p. 94 °C.

### Crystal data

**Table:**

C_11_H_11_F_1_O_2_	*F*_000_ = 408
*M**_r_* = 194.20	*D*_x_ = 1.287 Mg m^−^^3^
Monoclinic, *P*2_1_/*c*	Mo *K*α radiation radiation λ = 0.71073 Å
Hall symbol: -P 2ybc	Cell parameters from 2981 reflections
*a* = 4.1895 (4) Å	θ = 2.3–30.6º
*b* = 17.4362 (19) Å	µ = 0.10 mm^−^^1^
*c* = 13.8134 (15) Å	*T* = 296 (2) K
β = 96.719 (3)º	Prismatic, colourless
*V* = 1002.12 (18) Å^3^	0.25 × 0.18 × 0.12 mm
*Z* = 4	

### Data collection

**Table:**

Bruker KappaAPEXII CCD diffractometer	2981 independent reflections
Radiation source: fine-focus sealed tube	1704 reflections with *I* > 2σ(*I*)
Monochromator: graphite	*R*_int_ = 0.026
Detector resolution: 7.2 pixels mm^-1^	θ_max_ = 30.6º
*T* = 296(2) K	θ_min_ = 2.3º
ω scans	*h* = −5→6
Absorption correction: multi-scan(SADABS; Bruker, 2005)	*k* = −24→23
*T*_min_ = 0.935, *T*_max_ = 0.958	*l* = −19→19
8632 measured reflections	

### Refinement

**Table:**

Refinement on *F*^2^	Secondary atom site location: difference Fourier map
Least-squares matrix: full	Hydrogen site location: inferred from neighbouring sites
*R*[*F*^2^ > 2σ(*F*^2^)] = 0.050	H atoms treated by a mixture of independent and constrained refinement
*wR*(*F*^2^) = 0.164	*w* = 1/[σ^2^(*F*_o_^2^) + (0.0731*P*)^2^ + 0.1527*P*] where *P* = (*F*_o_^2^ + 2*F*_c_^2^)/3
*S* = 1.04	(Δ/σ)_max_ < 0.001
2981 reflections	Δρ_max_ = 0.41 e Å^−^^3^
131 parameters	Δρ_min_ = −0.21 e Å^−^^3^
Primary atom site location: structure-invariant direct methods	Extinction correction: none

### Special details

**Table:**

Geometry. All e.s.d.'s (except the e.s.d. in the dihedral angle between two l.s. planes) are estimated using the full covariance matrix. The cell e.s.d.'s are taken into account individually in the estimation of e.s.d.'s in distances, angles and torsion angles; correlations between e.s.d.'s in cell parameters are only used when they are defined by crystal symmetry. An approximate (isotropic) treatment of cell e.s.d.'s is used for estimating e.s.d.'s involving l.s. planes.
Refinement. Refinement of *F*^2^ against ALL reflections. The weighted *R*-factor *wR* and goodness of fit *S* are based on *F*^2^, conventional *R*-factors *R* are based on *F*, with *F* set to zero for negative *F*^2^. The threshold expression of *F*^2^ > σ(*F*^2^) is used only for calculating *R*-factors(gt) *etc*. and is not relevant to the choice of reflections for refinement. *R*-factors based on *F*^2^ are statistically about twice as large as those based on *F*, and *R*- factors based on ALL data will be even larger.

### Fractional atomic coordinates and isotropic or equivalent isotropic displacement parameters (Å^2^)

**Table:**

	*x*	*y*	*z*	*U*_iso_*/*U*_eq_	
F1	0.5705 (5)	0.28288 (7)	0.22076 (10)	0.1113 (6)	
O1	1.0669 (3)	0.08331 (7)	0.07503 (9)	0.0575 (4)	
H1	1.104 (5)	0.0617 (11)	0.0126 (15)	0.069*	
O2	0.8191 (3)	−0.02692 (7)	0.09434 (9)	0.0609 (4)	
C1	0.8986 (4)	0.03770 (8)	0.12367 (11)	0.0406 (4)	
C2	0.8045 (3)	0.06721 (8)	0.21699 (11)	0.0394 (4)	
C3	0.8530 (4)	0.14131 (9)	0.23791 (12)	0.0443 (4)	
H3	0.9555	0.1699	0.1938	0.053*	
C4	0.7616 (4)	0.18258 (9)	0.32326 (11)	0.0449 (4)	
C5	0.6226 (5)	0.25413 (10)	0.31195 (13)	0.0593 (5)	
C6	0.5260 (6)	0.29671 (11)	0.38672 (16)	0.0705 (6)	
H6	0.4286	0.3443	0.3749	0.085*	
C7	0.5763 (6)	0.26755 (11)	0.47922 (15)	0.0659 (6)	
H7	0.5120	0.2953	0.5311	0.079*	
C8	0.7214 (5)	0.19750 (12)	0.49543 (14)	0.0691 (6)	
H8	0.7586	0.1781	0.5585	0.083*	
C9	0.8126 (5)	0.15554 (11)	0.41831 (13)	0.0601 (5)	
H9	0.9105	0.1081	0.4304	0.072*	
C10	0.6348 (4)	0.01081 (9)	0.27580 (12)	0.0458 (4)	
H10A	0.4779	−0.0170	0.2319	0.055*	
H10B	0.5185	0.0392	0.3209	0.055*	
C11	0.8524 (5)	−0.04706 (11)	0.33354 (14)	0.0614 (5)	
H11A	0.7248	−0.0808	0.3682	0.092*	
H11B	1.0041	−0.0205	0.3791	0.092*	
H11C	0.9657	−0.0764	0.2897	0.092*	

### Atomic displacement parameters (Å^2^)

**Table:**

	*U*^11^	*U*^22^	*U*^33^	*U*^12^	*U*^13^	*U*^23^
F1	0.2170 (19)	0.0646 (8)	0.0569 (8)	0.0505 (10)	0.0350 (9)	0.0161 (6)
O1	0.0854 (9)	0.0450 (7)	0.0468 (7)	−0.0124 (6)	0.0275 (6)	−0.0072 (5)
O2	0.0912 (10)	0.0434 (7)	0.0528 (7)	−0.0149 (6)	0.0288 (7)	−0.0118 (5)
C1	0.0485 (8)	0.0360 (8)	0.0382 (8)	0.0015 (6)	0.0091 (7)	0.0007 (6)
C2	0.0426 (8)	0.0401 (8)	0.0357 (7)	0.0046 (6)	0.0061 (6)	0.0003 (6)
C3	0.0549 (9)	0.0404 (8)	0.0382 (8)	0.0029 (6)	0.0085 (7)	−0.0005 (6)
C4	0.0575 (10)	0.0386 (8)	0.0393 (8)	0.0012 (7)	0.0088 (7)	−0.0041 (6)
C5	0.0947 (14)	0.0413 (9)	0.0438 (10)	0.0086 (9)	0.0156 (9)	0.0029 (7)
C6	0.1047 (17)	0.0438 (10)	0.0653 (13)	0.0165 (10)	0.0194 (12)	−0.0084 (9)
C7	0.0898 (15)	0.0588 (12)	0.0521 (11)	0.0015 (10)	0.0203 (10)	−0.0183 (9)
C8	0.0999 (16)	0.0685 (13)	0.0391 (9)	0.0122 (11)	0.0095 (10)	−0.0042 (9)
C9	0.0866 (14)	0.0525 (10)	0.0406 (9)	0.0170 (9)	0.0047 (9)	−0.0027 (8)
C10	0.0478 (9)	0.0460 (9)	0.0456 (9)	−0.0026 (7)	0.0136 (7)	−0.0018 (7)
C11	0.0694 (12)	0.0513 (10)	0.0654 (12)	−0.0014 (8)	0.0163 (10)	0.0179 (9)

### Geometric parameters (Å, °)

**Table:**

F1—C5	1.350 (2)	C6—H6	0.9300
O1—C1	1.3006 (18)	C7—C8	1.371 (3)
O1—H1	0.97 (2)	C7—H7	0.9300
O2—C1	1.2301 (18)	C8—C9	1.383 (2)
C1—C2	1.483 (2)	C8—H8	0.9300
C2—C3	1.334 (2)	C9—H9	0.9300
C2—C10	1.506 (2)	C10—C11	1.521 (2)
C3—C4	1.470 (2)	C10—H10A	0.9700
C3—H3	0.9300	C10—H10B	0.9700
C4—C5	1.378 (2)	C11—H11A	0.9600
C4—C9	1.388 (2)	C11—H11B	0.9600
C5—C6	1.371 (3)	C11—H11C	0.9600
C6—C7	1.368 (3)		
			
C1—O1—H1	112.0 (12)	C6—C7—H7	120.0
O2—C1—O1	122.15 (14)	C8—C7—H7	120.0
O2—C1—C2	120.97 (14)	C7—C8—C9	120.19 (18)
O1—C1—C2	116.88 (13)	C7—C8—H8	119.9
C3—C2—C1	118.35 (14)	C9—C8—H8	119.9
C3—C2—C10	125.80 (14)	C8—C9—C4	121.56 (17)
C1—C2—C10	115.63 (13)	C8—C9—H9	119.2
C2—C3—C4	126.91 (15)	C4—C9—H9	119.2
C2—C3—H3	116.5	C2—C10—C11	115.09 (14)
C4—C3—H3	116.5	C2—C10—H10A	108.5
C5—C4—C9	115.53 (15)	C11—C10—H10A	108.5
C5—C4—C3	119.92 (15)	C2—C10—H10B	108.5
C9—C4—C3	124.53 (14)	C11—C10—H10B	108.5
F1—C5—C6	118.18 (17)	H10A—C10—H10B	107.5
F1—C5—C4	117.52 (16)	C10—C11—H11A	109.5
C6—C5—C4	124.27 (18)	C10—C11—H11B	109.5
C7—C6—C5	118.38 (18)	H11A—C11—H11B	109.5
C7—C6—H6	120.8	C10—C11—H11C	109.5
C5—C6—H6	120.8	H11A—C11—H11C	109.5
C6—C7—C8	120.03 (18)	H11B—C11—H11C	109.5
			
O2—C1—C2—C3	−169.70 (16)	C3—C4—C5—C6	179.3 (2)
O1—C1—C2—C3	10.1 (2)	F1—C5—C6—C7	179.6 (2)
O2—C1—C2—C10	5.2 (2)	C4—C5—C6—C7	1.5 (4)
O1—C1—C2—C10	−174.93 (14)	C5—C6—C7—C8	0.3 (4)
C1—C2—C3—C4	176.46 (15)	C6—C7—C8—C9	−1.0 (4)
C10—C2—C3—C4	2.1 (3)	C7—C8—C9—C4	0.1 (3)
C2—C3—C4—C5	−136.30 (19)	C5—C4—C9—C8	1.5 (3)
C2—C3—C4—C9	45.5 (3)	C3—C4—C9—C8	179.80 (19)
C9—C4—C5—F1	179.57 (19)	C3—C2—C10—C11	−106.93 (19)
C3—C4—C5—F1	1.2 (3)	C1—C2—C10—C11	78.57 (19)
C9—C4—C5—C6	−2.4 (3)		

### Hydrogen-bond geometry (Å, °)

**Table:**

*D*—H···*A*	*D*—H	H···*A*	*D*···*A*	*D*—H···*A*
O1—H1···O2^i^	0.97 (2)	1.66 (2)	2.6325 (18)	177.7 (12)
C3—H3···O1	0.93	2.32	2.713 (2)	105
C6—H6···O2^ii^	0.93	2.53	3.421 (2)	160
C8—H8···F1^iii^	0.93	2.55	3.266 (2)	134 (1)

Symmetry codes: (i) −*x*+2, −*y*, −*z*;  (ii) −*x*+1, *y*+1/2, −*z*+1/2; (iii) *x*, −*y*+1/2, *z*+1/2.
